# Perceptions of quality in primary health care: perspectives of patients and professionals based on focus group discussions

**DOI:** 10.1186/1471-2296-15-128

**Published:** 2014-06-28

**Authors:** Renata Papp, Ilona Borbas, Eva Dobos, Maren Bredehorst, Lina Jaruseviciene, Tuulikki Vehko, Sandor Balogh

**Affiliations:** 1 National Institute of Primary Health Care, 84-88 Jász Str, Budapest 1135, Hungary; 2 National Institute for Quality- and Organizational Development in Health Care and Medicines, 1 Hold Str, Budapest 1054, Hungary; 3 University of Bielefeld, 25 Universitäts Straße, Bielefeld 33615, Germany; 4 Lithuanian University of Health Sciences, 2 Eivenių, Kaunas 50009, Lithuania; 5 National Institute for Health and Welfare, 166 Mannerheimintie, Helsinki, Finland

**Keywords:** Quality, Primary health care, Patient satisfaction, Gate-keeping

## Abstract

**Background:**

The EUprimecare project-team assessed the perception of primary health care (PHC) professionals and patients on quality of organization of PHC systems in the participating countries: Estonia, Finland, Germany, Hungary, Italy, Lithuania and Spain. This article presents the aggregated opinions, expectations and priorities of patients and professionals along some main dimensions of quality in primary health care, such as access, equity, appropriateness and patient- centeredness.

**Methods:**

The focus group technique was applied in the study as a qualitative research method for exploration of attitudes regarding the health care system and health service. Discussions were addressing the topics of: general aspects of quality in primary health care; possibilities to receive/provide PHC services based on both parties needs; determinant factors of accessibility to PHC services; patient centeredness. The data sets collected during the focus group discussions were evaluated using the method of thematic analysis.

**Results:**

There were 14 focus groups in total: a professional and a patient group in each of the seven partner countries. Findings of the thematic analysis were summarized along the following dimensions: access and equity, appropriateness (coordination, continuity, competency and comprehensiveness) and patient centeredness.

**Conclusions:**

This study shows perceptions and views of patients in interaction with PHC and opinion of professionals working in PHC. It serves as source of criteria with relevance to everyday practice and experience. The criteria mentioned by patients and by health care professionals which were considered determining factors of the quality in primary care were quite similar among the investigated countries. However, the perception and the level of tolerance regarding some of the criteria differed among EUprimecare countries. Among these dissimilar criteria we especially note the gate-keeping role of GPs, the importance of nurses' competency and the acceptance of waiting times. The impact of waiting time on patient satisfaction is obvious; the influence of equity and access to PHC services are more dependent on the equal distribution of settings and doctors in urban and rural area. Foreseen shortage of doctors is expected to have a substantial influence on patient satisfaction in the near future.

## Background

“Primary Health Care: Now more than ever”, says the WHO in its 2008 report [1]. There is no longer any doubt that the cost-efficiency of health care services can be improved by shifting the emphasis towards primary health care (PHC). However, there is a scarcity of data concerning the relative effectiveness of different organizational models; each European country tries to find its own recipe of better ways to deliver primary health care, which is essential for long term sustainability of mainly publicly funded health care.

The provision of health care services can be considered the sum of doctor-patient interactions which occur in an organizational and social context and within a system of infrastructure [2]. Then, the assessment of quality goes beyond the estimation of the professional standards’ application in practice [3,4]. In order to understand the quality of health care services provided, it is necessary to assess views from more than one perspective, finding the right balance between the views of patients (customers) and different health care professionals (service providers).

Patients are in the best position to evaluate their experience of care, but some studies raise a doubt whether this experience could be a good measure of effectiveness of care [5]. Research indicates that patients’ perception of quality is influenced by a variety of factors, such as features of the national health system [6,7], practice type [8] and the providers’ personal and clinical skills [9]. It seems that patients value immediate comfort [5] while physicians addressing quality of care are concerned more about resources [10,11]. However, practitioners and patients have, to some extent, a shared position on quality of care [12] and their apparently diverging perspectives, in fact, could be understood as complementary.

A large body of research addressing different aspects of quality of care from the perspective of patients and providers exists. However, these studies mostly cover a single country [12-14] and/or one health problem [15-17]. There is a lack of data about the perception of quality of care by patients and health providers in an international context.

The health benefits of health system organization and delivery characteristics are still not well recognized, despite the efforts of the past 10 years at health services reform [18].

To improve the health status of the population and to respond to people’s expectations about their health, it is essential to have a sound understanding of the relationship between the specific characteristics of Primary Care and its outcomes. The EC funded EUprimecare [19] project aimed to provide evidence of the links between quality of care and its cost in Primary Care through a set of research methods and tools. The EUprimecare project analyzed variations of both quality and costs as related specifically to organizational models to uncover possible trade-offs between quality and costs in each model. An important part of the EUprimecare research had the objective of developing a set of clinical indicators identified by literature review and a set of organizational indicators based on the selected set of criteria identified through perceptions of patients and professionals in PHC. The current article will present the results of focus group discussions performed to identify quality criteria considered relevant by groups of patients using PHC services and professionals providing PHC services.

Focus group discussions were performed in the countries involved in the EUprimecare [19] project consortium: Estonia, Finland, Germany, Hungary, Italy, Lithuania and Spain. The selection of the countries tried to cover a variety of health care systems in the European Union (EU) differentiated by their financing procedures: taxation–based (for example Spain, Finland) or insurance-based systems (for example Germany, Estonia); by centralization of the provision of health care services: those with a mainly centralized health care system (for example Hungary, Lithuania, Germany) or decentralized (for example Italy and Spain); by the gate-keeping role of primary health care: a 100% gate-keeping role (for example Lithuania) and partial gate-keeping (for example Estonia, Hungary). As a first step of the EUprimecare project, the models of provision of primary health services were described. Construction of the models started with basic observations of how those health services that are usually regarded as PHC are configured in different health systems. The following models have been identified [19]:

PHC based on choice of specialists’ services

PHC based on individual generalists

PHC based on group practices

PHC through health centers

PHC through integrated services

Table 1 summarizes the features of PHC identified in the participating countries.

**Table 1:** Basic characteristic of PHC in the EUprimecare countries

	**Hungary**	**Italy**	**Spain**	**Lithuania**	**Finland**	**Estonia**	**Germany**
**Model type**	**PHC based on individual generalists**	**PHC based on individual generalists**	**PHC based on individual generalists and PHC based on group practices**	**PHC based on group practices**	**PHC through health centers**	**PHC through health centers**	**PHC based on choice of specialists’ services**
Model homogenity	Mainly solo practices	75% solo practices	40% solo practices, 60% group practices	75% group practices	100% health centers	100% health care centers	Mainly ambulatory care specialists in solo practices and some polyclinics
PC practice ownership	Private	Private	Private	75% public, 25% private	98% public	Public	Solo practices are private
Employment type of GP	Private enterpreneurs	Private enterpreneurs	Private enterpreneurs	Mostly employees	Employees	Mostly employees	Private entrepreneurs in practices and employed professionals in policlinics
	Hungary	Italy	Spain	Lithuania	Finland	Estonia	Germany
Payment methods	Capitation and some extra on the basis of the practice characteristics, P4P scheme based on quality indicators	Capitation	Capitation (73%), fees for services (15%), basic allowance (10%), other (2%)	Capitation (85%), fee for service (9%), bonus for the performance (6%)	Salary and additional fee for service, and bonuses for performance.	Salary and capitation (15%)	Mixture of fees per time period and per medical procedure
Gatekeeper for referrals	Yes	Yes	Yes	Yes	Yes	Yes	Not characteristic, but national incentives promote the gategeeping role of GPs

With the objective of developing indicators to assess the level of organization of PHC, relevant quality dimensions were considered. Quality dimensions are definable, measurable and actionable attributes of the quality of care [20]. The domains of access to first contact care, coordination, comprehensiveness, community orientation, and family orientation [21,22] are the recognized structural bases of the primary care process, and the ones which are associated with quality of services [23], patient satisfaction, effectiveness, efficiency and equity [24]. This article presents the aggregated opinions, expectations and priorities of patients and professionals along the dimension of quality in primary health care, such as access, equity, appropriateness and patient centeredness. Longitudinality, coordination, comprehensiveness were assessed in the frame of appropriateness; community orientation, and family orientation in the frame of patient centeredness.

## Methods

The focus group technique [25,26] was applied in the study as a qualitative research method for exploration of attitudes regarding the health care system and health service. There were two types of focus groups involving patients and primary health care professionals separately. Each group consisted of 8 to 10 people, which represents the ideal size of a focus group.

The focus groups were coordinated by a leading team from Hungary. There were 14 target focus groups in total: a professional and a patient group in each of the seven partner countries. The exact number of participants is indicated in Tables 2 and 3.

**Table 2:** Demographic breakdown of focus group participants – health professionals

**Indicator**	**Estonia**	**Finland**	**Germany**	**Hungary**	**Italy**	**Lithuania**	**Spain**	**Total**
**Gender**								
Male	1	-	4	2	3	3	4	17
Female	9	6	5	6	7	8	6	47
**Age (years)**		NA						
<31	-		-	-	-	1	-	
31-40	2		-	1	1	4	3	
41-50	3		3		3	4	3	
51-60	5		4	7	6	2	4	
>60	-		2	-	-	-	-	
**Occupation**								
GP	6	3	5*	5	3	5	5	32
Pediatrician	-	-	1	1	3	2	1	8
Internist	-	-	-*	-		2	-	2
Gynaecologist or other specialist	-	-	3	-	1	-	-	4
Nurse	4	3	-	2	3	2	4	18
**Total**	**10**	**6**	**9**	**8**	**10**	**11**	**10**	**64**

*predominantly internists registered as GP.

**Table 3:** Demographic breakdown of focus group participants - patients

**Indicator**	**Estonia**	**Finland**	**Germany**	**Hungary**	**Italy**	**Lithuania**	**Spain**	**Total**
**Gender**								
Male	4	4	4	3	5	4	3	27
Female	3	4	4	8	4	5	6	34
**Age (years)**		NA						
<31	1		-	2	2	3	1	
31-40	1		1	3		1	1	
41-50	1		3	2	2	1	2	
51-60	1		3	2	2	2	2	
>60	3		1	2	3	2	3	
**Education**		NA						
Secondary, or vocational training	5		7	3	3	3	7	
Higher degree	2		1	8	6	6	2	
**Total**	**7**	**8**	**8**	**11**	**9**	**9**	**9**	**61**

### Participants

In order to facilitate free discussion and to approach as many perspectives as possible, participants of the focus groups were chosen carefully according to the following selection criteria. Selection criteria for primary health care professionals included: working with patients on a daily basis, involvement of physicians and nurses, and equal distribution based on age group, gender, and practice in urban/rural areas. Professionals were recruited in each country at Continuous Medical Education (CME) conferences organized for PHC professionals. Recruitment of patients was organized in a neutral area from the perspective of PHC setting in order to avoid bias caused by the closeness of getting these services, but in a place with a high probability of finding patients who have visited a PHC provider in the last 12 months (for example: pharmacies or out-patient clinics working in a separate building, but accessible with referral). Criteria for patients were: age, gender, education level, type of residence and usage of any PHC service in the past 12 months. The details of 40-50 participants satisfying the selection criteria for each group were entered into a database. A database algorithm randomly subselected members of each group who were then contacted and invited to join the focus group discussion. The demographic breakdown of the participants is visualized in the attached tables (professionals - Table 1; patients - Table 2). Ethical approval was obtained in each partner country (in Estonia by the Research Ethics Committee of the University of Tartu; in Finland by the Ethics committee of the National Institute for Health and Welfare; in Germany by the Ethics Committee of the Medical Chamber of Westfalia-Lippe and the Medical School of Münster University; in Hungary by the Ethical and Research Committee of Health Science Council; in Italy by the Legal Affairs Office of University of Bocconi; in Lithuania by the Bioethics Committee of Lithuanian University of Health Sciences, in Spain by the Ethical and Research Committee of Instituto de Salud Carlos III) and informed consent documents were signed by each participant.

### Running focus group discussions

Focus group discussions (FGD) were run by a facilitator with the responsibility to apply the appropriate working group techniques. The facilitator was required to provide equal opportunities for communication to all subgroups of patients and professionals. The facilitator did not act as an expert but stimulated and supported discussion addressing the topics of:

– general aspects of quality in primary health care

– possibilities to receive/provide PHC services based on both parties needs

– determinant factors of accessibility to PHC services

– patient/professional experience of patient-focused PHC-service provision (ie. patient centeredness)

Focus group discussions lasted for about 1.5 - 2 hours. Each focus group discussion was audio-taped and verbatim transcribed.

### Data analysis

The data sets collected during the focus group discussions were evaluated using the method of thematic analysis [27]. The contents of the recorded discussions were codified into different themes according to preset criteria. The first list of codes was defined during pilot focus group discussions performed in Hungary, one among patients and one among professionals. The set of quality criteria were identified based on the literature [28], then used as codes to aggregate the transcript of the discussions. The initial list of codes was accepted by the research team of the EUprimecare project. Each code (quality criteria) had to have a supporting sentence, extracted from the FGD transcript and a relevant comment added by the evaluator. New codes were added as required based on the emerging transcripts.

The facilitators took notes of the respondent characteristics; influence by other participants; context within which the comments were made; internal consistency - for example changes in opinion or influence by other participants; frequency and extensiveness; specificity of comments, such as personal experience or hypothetical situation; intensity of comments, like depth of feeling; relationship with other criteria. Therefore, in the analysis of individual opinions, the opinions that changed due to group dynamics, as well as the opinions of groups expressed on the basis of consensus, were identified.

## Results

There were two focus groups organized in each of the seven countries, one for patients and one for health care professionals, so in total 14 transcripts were analysed.

The identified codes were organized along the following dimensions (defined at the beginning of the study): access, equity, appropriateness, patient and professional satisfaction.

The following meanings were considered for the dimensions chosen:

– Access is the ease with which health services are reached [29].

– Equity defines the extent to which a system deals fairly with all concerned. Equity, in this context, deals with the distribution of health-care and its benefits among the population.

– Appropriateness: doing the right things, with the right knowledge and the right skills, in the right context, in the right place and at the right time [30]. Accordingly, appropriateness includes:

– Competency/skills: the degree to which health personnel are trained and able to assess, treat and communicate (listening, patient education and sharing decisions) with their patients [28,31].

– Comprehensiveness refers to the wide range of services comprising curative care, rehabilitation and supportive care, as well as health promotion and disease prevention [32].

– Coordination/continuity: the extent to which health-care for specified users is coordinated across providers and institutions over time [28,31].

– Patient centeredness is the degree to which a system actually functions by placing the patient at the center of its delivery of health-care and is often assessed in terms of the patient’s experience [28,30,31]. Family- and community- orientation were also included under this dimension.

For each partner country FGDs were analyzed separately for each target group (patients/professionals) presented as a code map, and summary, and then compiled.

Compilation of the summaries sent by each country are presented along the dimensions separately for the two target groups: patients and PHC professionals

– Access and equity

– Appropriateness (coordination, continuity, competency and comprehensiveness)

– Patient centeredness.

Criteria listed in the code map, indicating that they were mentioned during the discussions in one of the focus groups, are presented in Table 4.

**Table 4:** Criteria listed in the code map

**Dimensions/Criteria**	**How addressed during discussion**
**APPROPRIATENESS**	
**Competency/skill**	Professional training
	Continuous medical education
	Competency in PHC practice/services
	Gate-keeping
**Comprehensiveness**	Preventative services
	Long-term care for chronic condition
	Provisiond of other non-medical services (social services)
	Holistic approach
**Coordination/continuity**	Usual source of care (first contact with new health problems, care for the majority of health problems)
	Long-term follow-up
	Patient record continuity
	Referral process between PHC and specialist
**Professional decision making procedure**	Use of evidence based practice guidelines
	Involvement of patients
**Timeliness**	Classification of cases by urgent needs
**Effectiveness**	Improving of health status
	Minimalisation of unnecessary visit
	Provision of care is adapted to practice setting and enviroment
**Safety**	Information safety
	Reporting critical incidents
	Infection control
	Care without mistakes
**Practice management**	Medical equipment
	Non-medical eqiupment
	Quality management tools
**ACCESSIBILITY - EQUITY**	
**Geographical access**	Access via telecommunication tools
	Access in time (office hours, length of one visit)
**Availibility in time; staff**	Appointment system
	Waiting time
**DIMENSIONS/CRITERIA**	**How addressed during discussion**
**ACCESSIBILITY - EQUITY**	
**Availibility in time; staff**	Capacity of human resources in the practices
	Home visits
**Equity**	Financial constrains
	Provision services to people in different age groups
	Provision services to people at risk of social exclusion
	Provision services to disabled people
**PATIENT CENTEREDNESS**	
**Communication skills**	Patient/family education with reference to adherence
**Interpersonal attributes**	Kindness of staff
**Privacy/confidentiality**	Privacy of the visit
	Privacy of patient information
**Acceptability**	Comfort of the waiting room, conditions of the premises
**Community oriented health care**	Community based programs

### Access and equity

Geographical access was ranked as an important aspect of the quality of primary care, among patients and professionals alike.

“Access to treatment is an elementary part of high quality health care” (Finnish patient).

In the opinion of professionals the fair geographical distribution of resources and practices is an important aspect in guaranteeing access to PHC. In the majority of countries - due to different distances, infrastructure and availability of public transport - there are differences in access to primary care between urban and rural areas.

Accessibility in time*,* namely the shortest possible time to reach primary care services was found to be another important aspect. Patients wish to reach their GPs immediately when they need them, meaning quick and easy access with short waiting time. Such high patient expectation leads to frustration among professionals.

“Every time you go to see your doctor, he should receive you!” (Italian patient).

“They [patients] would like physicians to be always available. They wish to visit their GP whenever they want all day long, to call him any time, the phone would never be occupied, the physician would never be sick, would never have leisure time, etc… They imagine that the physician should be dedicated to his work and cannot be sick, have vacations or bring up his children; this is the point of view of the patients… This is bad; in fact, we need to educate people…” (Lithuanian family physician).

In consequence, patients often think that the appointment system is a limiting factor for their access to the PHC system; it postpones consultation to a time later than the time of their need. Nevertheless, patients in different countries seem to have different tolerance levels regarding waiting times for GP care. While for a Finnish patient even one week of waiting time is acceptable in a non-acute case, in other countries, like Hungary, Lithuania and Spain any waiting that restricts immediate care is negatively perceived.

Differences in opinion between patients and professionals were found regarding home visits, too, which concern mainly patients' sense of comfort and safety. Patients expect the possibility of home visits more often than the doctor feels it is necessary.

“A GP/paediatrician is considered a good doctor when he does home visits, but in my opinion this is wrong: we should go to see our patients at home only if it is strictly necessary”. (Italian professional)

In some countries, patients need to contact their GP by mobile phone, thus ensuring continuous accessibility.

“Now it is excellent when you can call the physician. My sister had a problem on New Year's eve, she got a flu. She lives alone, so she called the GP, talked it over with her and it was very good”. (Lithuanian patient)

Doctors, by contrast, emphasize that it is the office that patients should have access to, and the out-of-hours service is available to them after consulting hours if needed. German patients would regard it as a rare privilege to reach their GP out of office hours, but at the same time they are easily upset if they cannot get through to the official practice line.

“We are not obliged to give our mobile numbers: patients can call our offices and there are other services (e.g. duty doctors) during the weekends”. (Italian professional)

GPs would also need concrete protocols for phone consultations in order to avoid mistakes. One Estonian professional argued that consultation without seeing the patient is not safe.

The level of equity can be influenced by geographical characteristics, social factors (e.g. the elderly, without family support, can have lower access to PHC services) and economic differences as well. These last two factors are linked to the health insurance coverage and reimbursement of health care services. Even if the coverage rate among the population is high, out-of-pocket payments for additional services cause uncertainties and are perceived as inequities:

“…and now it's become like this (with the preventive examinations), you have to pay them from your pocket, but that's not possible for everyone, some people just do not have the money”. (German patient)

Active discrimination in PHC services was not reported by either patients or professionals.

### Appropriateness

#### *Coordination, continuity*

Patients from all of the countries involved in the study had a strong need for the continuity of primary care and pointed out the importance of long standing personal relationships with their physician. Patients need their doctor to coordinate their care and the services provided by other professionals, in other settings and at other levels of the health care system.

“It is important that one doctor sees the whole process of the illness. So the patient should not tell another doctor the whole case history again and again”. (Hungarian patient)

The exchange of information between primary and secondary care is commonly perceived as insufficient:

“In my opinion, there is a lack of coordination, and sometimes people may fall into despair. You have to wait a while, then they send you to the specialist, and finally the specialist sends you back to the general practitioner again for treatment or …, and the GP says: well, I can’t do it”. (Spanish patient)

It was suggested that the GPs should have access to information from secondary care through the IT system. Group practices, particularly joint practices of GPs with specialists were considered advantageous.

“*Whenever I have to see one of them, they are in the same building.//So you are referred directly?//yeah, just go upstairs, that's it. I get a referral slip and I'm there, it's quick”. (German patient)*

“The quality of continuity of care for chronic diseases is guaranteed by the existence of a professional team, where everyone has a specific and clear role: to be professionals of good quality, GPs need to work in an organised group”. (Italian professional)

Good communication skills and appropriate regulations were both considered important coordination tools. However, the opinion of professional groups corroborates patient statements concerning the lack of coordination and information exchange between different actors of the health care system - being dependent on personal factors rather than regulations:

“The coordination between primary care professionals and specialists is based on our personal goodwill”. (Italian professional)

In the countries where it is applicable, the gate-keeping role of the family doctor is interpreted by patients as an unnecessary barrier to reaching a specialist.

“It seems comical – I have chronic eye disease, but before addressing the eye specialist I should go to the GP, to wait in a queue just to get the referral”.(Lithuanian patient)

#### *Competency, comprehensiveness*

“GPs are required to solve the patients’ health problems” – in this way patients formulate the core competence of family physicians:

“Competency means effective problem solving”. (Estonian patient)

Professionals emphasized the following competencies of primary care: solving patients’ problems adequately, making patients understand their health status and making them compliant to the therapy. These require having enough time to talk to patients in order to get to a proper anamnesis and work out therapeutic plans together with the patient.

“Our duty is to treat our patients in the most practical way, trying to be as close as possible to our patients - that is why they call us “family doctors”. (Italian professional)

In almost every country GPs are expected to perform preventative activities on top of curative ones.

“The family doctor should not only treat you, but also help you to live a healthy life, giving suggestions on how to prevent diseases”. (Italian patient)

Despite some good examples mentioned in different countries, it was brought up that doctors are not sufficiently engaged in prevention and patients do not show enough interest in preventative services.

“In my experience, doctors have never asked me anything else but the reason for coming”. (Finish patient)

“You should go to the physician when you feel bad or when it hurts. If everything is OK, I do not know whether we should go […]. Since my youth I go to the doctor only when I feel bad. We should go when it is very bad. This is my opinion”. (Lithuanian patient)

Similarly to patients, professionals think that prevention and patient education are important tasks of primary care, even though they do not receive sufficient attention as resources are directed towards reimbursement of acute disease cases. This may explain some patients' perception that doctors do not like to spend their time with prevention for reasons of economic benefit.

“In patient education, we should rely more on well-trained health care professionals, but it is not financed”. (Hungarian professional)

GPs and nurses feel in charge of the education of the population on health issues and of improving health literacy, but they are also aware of the economic and organizational constraints of their work. They think that this education process needs to be supported by the overall health care system and the education system:

“Motivating people takes a lot of time. Time and much stress. In the area of prevention: if you sit down with a patient and talk about his smoking behavior for example, it takes you 20 or 30 minutes, and then you have actually achieved something. But this is not being rewarded financially, which is very, very unsatisfying” .(German professional)

“It is common that a patient tells the doctor what kind of problems he had and what kind of symptoms he had, but it is also the other way around after the diagnosis the doctor should tell in very clear words about the self-treatment and treatment scheme in the secondary care”. (Finish patient)

Administrative tasks are perceived as an especially heavy burden by professionals.

In addition, it is prevalent in all countries that a significant proportion of patients visit their GP with social problems - medical and social aspects of care being closely interrelated.

*“In an epidemic period, 80*% *of the population goes to the doctor because of illness, 20*% *because of a problem. When there is no epidemic, the situation is reverse. Beyond acute and chronic problems, patients come to us because of social problems, too… Primary care is close to the population and the patients think that they can go to primary care consultation with any kind of problem”. (Hungarian professional)*

Regarding professional decision making, health professionals underlined the importance of following evidence-based clinical guidelines and practice protocols. However the balance between guidelines and personalized care is taken into consideration.

“*Professional decision making is 50 percent guideline, 50 percent agreement with the patient”. (Hungarian professional)*

“I used to have a check-up of current care guidelines before an unusual patient case”. (Finnish professional)

Besides professional motives, decision making is also influenced by economic considerations.

“I would differentiate between patients’ needs and legitimate needs, i.e. what our system has to offer and reimburses for the patients”. (German professional)

In the majority of focus groups, the opinions of patients and doctors coincide regarding the importance of nurses in primary health care. There are differences, however, among particular countries in judging the nurse’s role. In Finland, for example, the role of nurses in primary care is strong. The tasks of nurses as seen by a Finnish professional are:

“The nurses’ job are the assessment of treatment need on the phone and during reception, the assessment of need for X-ray imaging, writing permission to be absent from work or school, wound treatment, the removal of stitching, anticoagulant treatment, blood pressure follow-up, assistance of the doctor, etc”. (Finnish professional)

While in the opinion of a Finnish patient:

“Experienced nurses guarantee the quality of treatment”. (Finnish patient)

In other countries, where the competences of nurses are not well established, patients are ambiguous about what they expect and accept from nurses.

“The nurse is not undertaking the big tasks as she is somehow not responsible for anything. Thus, she does what she is told to do, but does not take any initiative”. (Lithuanian professional)

“Nurses' work is undervalued and the nurse is perceived as not useful… The nurse is not appreciated by patients either, everybody says – “oh, nurse doesn’t know anything…” (Lithuanian professional)

### Patient centeredness

Patients expect the doctor to be empathic, friendly, attentive and sympathetic. Privacy and confidentiality as aspects of quality are rarely mentioned explicitly. Yet the relationship should be much more a partnership than a patriarchal connection. Patients expect to be listened to by their GPs.

“The ideal doctor-patient relationship is an empathic, discrete, consultative partnership. The doctor should treat the patient as a human being, not only as a disease”. (Hungarian patient)

Patients would like their GP to spend more time on the consultation and examination than is generally available in current practice. Appropriate communication contributes to a climate of confidence for the patients and thus to a good doctor-patient relationship. The information for patients should be understandable and clear, and the GP should spend time to explain the situation to his patients.

“Patients would feel that the doctor has now time for him/her and his/her issues (sometimes doctors are too busy)”. (Finish patient)

According to patients’ opinion, experienced nurses should have an important role in providing information to patients.

Among professionals certain personal characteristics are reported as a prerequisite for becoming a good GP: GPs must be attentive, friendly, comprehensive, good listeners, and must have willingness to help.

“The communication should be the basis of our job. To communicate is fundamental to make our patients aware of what a healthy life is. Primary care is based on communication”. (Italian professional)

However, with regard to the doctor-patient relationship, the doctors emphasized the importance of patients' responsible behavior more often than the patients themselves. The involvement of patients in the decisions about therapy is one of the noteworthy determinants of quality in the opinion of professionals.

“The quality level is determined by the patients. It is essential that all the patients are involved in the decision-making process”. (Spanish professional)

## Discussion

In order to fulfill the aims of the study and understand the attitudes and perceptions of patients and PHC professionals with regards to the quality dimensions of primary health care the qualitative method of focus group discussions was applied. Qualitative research methods are designed to help researchers understand people and what they say and do; such methods help to understand the social and cultural context of people's lives and actions. One of the key benefits of this research approach was the ability to observe and understand the context within which decisions and actions take place [33]. The “focus group discussion” technique was the qualitative research method employed. It facilitated free discussion; participants inspired each other to reveal past experiences, helped each other to express their views and catch the core content. The authors of this article considered focus group discussions more suitable than one-by-one interviews. In a group, the participants' subjective perspective can stimulate the group dynamic thus facilitating interactions and exchange of opinion [25,26].

Dimensions of quality, such as access, appropriateness and patient centeredness were considered for analysis.

The criteria mentioned by patients and by health care professionals which were considered determining factors of the quality in primary care were quite similar among the investigated countries. However, the perception and the level of tolerance regarding some of the criteria differed among EUprimecare countries. Among these dissimilar criteria we especially note the gate-keeping role of GPs, the importance of nurses’ competency and the acceptance of waiting times. Quantitative measurement of the impact of these factors and concrete differences were done in the frame of the project and will be presented in other articles.

Different interpretations among participants from countries supports the findings of other study which considers behavior of the population as important control knob of health care system, besides reimbursement, organization and payment mechanisms [34].

Geographical access is influenced by the distribution of primary health care settings, particularly in the context of urban–rural distribution. The number of doctors should be determined based on population needs. Accordingly, a reasonable distribution of practices between rural and urban areas should facilitate access to primary care services. A shortage of doctors is already being experienced in the EUprimecare monitored countries and is more accentuated in rural, deprived areas [35]. This study showed that there are differences in access to primary care between urban and rural areas in the majority of the participating countries due to differences in geographical distribution and public transport.

According to some estimates, Europe expects a shortage of one million health workers by 2020. Demand and supply of health workers is influenced by multiple factors: aging population, aging workforce, rising care-usage and rising costs within the context of budget constraints [36]. Primary health care professionals call for an increase in the number of doctors in the near future. A prospective solution to long-term GP shortages would be to assign some of the tasks currently performed by GPs over to nurses. This represents an opportunity to reshape general practice to meet the demands of the future [37]. So called task-shifting is a tool devised by the World Health Organization (WHO) to address the burden imposed by HIV/AIDS disease on the health care systems of developing countries. Such task-shifting expands the pool of human resources for health care provision by passing some competencies to personnel with a lower level of training. The WHO also stressed that task-shifting, although an efficient approach, will require significant investment and should not be seen as a substitute for other investments in human resources for health care [38]. If governments start to put in practice task-shifting in primary care, by giving extra competencies to nurses, they should bear in mind the complexity of family medicine [39]. During the focus group discussions there were already differences identified among countries in judging the nurse’s role. In Finland nurses take an important role in the management of treatment initiated by general practitioners, while in other countries nurses do not have such outlined competencies. This correlates with the different model of PHC in Finland, health center based compared with the solo practices found in Italy and Hungary (Table 4). Having a larger staff, health centers integrate the different competencies of professionals having influence on the acceptability by the patients.

The impact of waiting time on patient satisfaction is obvious [40]. An identified access-related need of the patients is the possibility of consultation by phone with their GP. On the other side, doctors, would need explicit guidelines for such phone consultations taking into account the risk of providing medical advice without face-to-face examination. Tele-consultation guidelines and reimbursement for such activities would improve professional satisfaction and patient safety.

Patients should have equal opportunities in their access to health care. In rural areas, far from specialized care, there is greater need for the management of care by GPs. However, the personality of the doctor contributes towards assuring equal opportunities to a greater extent than urban/rural location. This is another important example which demonstrates that the quality of primary care, at a patient and community oriented health care level, depends on the personality of the PHC provider. The GP's personality was also found to influence the level of coordination with secondary care.

Patients expressed a desire for the inclusion of various preventative activities amongst the range of services provided by their GP. However, GPs wish to be reimbursed for such services and suggest that preventative activities may be more effectively promoted at a national/EU level. Educating the public about health-promoting lifestyles and disease prevention at the national level would release limited GP resources to focus on actually treating patients’ illnesses.

According to the literature [41-43] regarding the relationship between the GP's gate-keeping role and patient satisfaction, there is a demonstrable correlation between patient satisfaction and the organizational aspects of GP service and also with the extent of patients' direct access to specialist care. This study showed clearly that patients are not enthusiastic about going to their GPs only to get a referral, especially if their chronic disease is managed at secondary care level.

During following steps of the EUprimecare project, criteria obtained based on the perceptions of the participants of the focus group discussions were transformed into quality indicators using literature references. There are widely accepted instruments for evaluating the organizational aspects of primary care. In Europe, the organizational indicators of primary care were established in 2002-2003 by the European Practice Assessment (EPA) project with the involvement of nine countries and with the support of expert panels [44]. The EPA project was concentrating on the measurement of the quality of a single practice, not of the whole system. Between 1995 and 1998 the revised form of the EUROPEP questionnaire, developed within the framework of an international consortium, enabled patients to evaluate the quality of primary care [45]. The results of EUprimecare’s focus group discussions presented in this article confirmed the relevance of the established EPA/EUROPEP indicators. The most important areas affecting the quality of primary care include access to care, patient-centeredness of care, clear definition of professional competencies and coordination of care.

### Limitations

The number of focus groups in each country (only two) was limited by budget constraints and that could have influenced the number of established criteria. This limitation was offset by conducting a literature research with the aim of including all known important aspects.

The number of participants in each focus group discussion was 8 to 10 people. A larger group would have limited the detail of some responses because participants feel a pressure to share airtime with others. Conversely, participants in a smaller group might feel an uncomfortable pressure to talk more than they would otherwise to fill dead air [46].

Although the perceptions of professionals on professional satisfaction were also addressed during focus group discussions, the present article did not consider this dimension, as the EUprimecare project team decided to discuss the results on this topic together with the results of the survey on primary health care professional satisfaction in a separate publication.

Primary health care is tasked with the treatment and prevention of chronic medical conditions in large, diverse populations. The broad scope of roles, and the size and diversity of the target populations, complicates our ability to evaluate PHC quality particularly when compared to the smaller, selected, populations observed in secondary health care.

## Conclusions

Very strict conclusions are difficult to be drawn from a qualitative analysis. However, this study shows, at international level, the perceptions and views of patients interacting with PHC and opinions of professionals working in PHC. It serves as a source of criteria with relevance to everyday practice and experience. Based on these criteria quality indicators can be found in the literature or developed, if necessary.

This study shows that the personality of the GP is a determinant of the quality of care but is difficult to be planned and influenced. From this point of view, those countries where PHC is provided by individual generalists are more vulnerable than those based on group practices or health centers.

The evolution of PHC models operated by different European countries has been guided over the long term by country-specific requirements. However, despite their differences, we have found that the shared challenges in PHC quality for the studied countries are access, equity, appropriateness, and organizational responsiveness to patient and professional needs.

## Competing interests

There are no competing interests applicable.

## Authors’ contributions

PR made substantial contributions to conception and design, acquisition and interpretation of data, and formulated the final format; IB contributed to data analysis; ED made substantial contributions in the development of the methodology; MB participated in the acquisition and interpretation of data, drafting the manuscript and revising it critically for important intellectual content; LJ was involved in the acquisition and interpretation of data, drafting the manuscript and revising it critically for important intellectual content; VT was involved in acquisition and interpretation of data, drafting the manuscript and revising it critically for important intellectual content; SB gave final approval of the version to be published. All authors read and approved the final manuscript.

## Pre-publication history

The pre-publication history for this paper can be accessed here:

http://www.biomedcentral.com/1471-2296/15/128/prepub
